# Predictability of US West Coast Ocean Temperatures is not solely due to ENSO

**DOI:** 10.1038/s41598-019-47400-4

**Published:** 2019-07-29

**Authors:** Antonietta Capotondi, Prashant D. Sardeshmukh, Emanuele Di Lorenzo, Aneesh C. Subramanian, Arthur J. Miller

**Affiliations:** 1 University of Colorado, Cooperative Institute for Research in Environmental Sciences, Boulder, CO USA; 2 NOAA, Earth System Research Laboratory, Physical Sciences Division, Boulder, CO USA; 3 Georgia Institute of Technology, Atlanta, GA USA; 4 University of Colorado, Boulder, CO USA; 5 Scripps Institution of Oceanography, University of California San Diego, La Jolla, CA USA

**Keywords:** Physical oceanography, Physics

## Abstract

The causes of the extreme and persistent warming in the Northeast Pacific from the winter of 2013/14 to that of 2014/15 are still not fully understood. While global warming may have contributed, natural influences may also have played a role. El Niño events are often implicated in anomalously warm conditions along the US West Coast (USWC). However, the tropical Pacific sea surface temperature (SST) anomalies were generally weak during 2014, calling into question their role in the USWC warming. In this study, we identify tropical Pacific “sensitivity patterns” that optimally force USWC warming at a later time. We find that such sensitivity patterns do not coincide with the mature SST anomaly patterns usually associated with ENSO, but instead include elements associated with ENSO SST precursors and SST anomalies in the central/western equatorial Pacific. El Niño events that produce large USWC warming, irrespective of their magnitude, do project on the sensitivity pattern and are characterized by a distinct evolution of the North Pacific atmospheric and oceanic fields. However, even weak tropical SST anomalies in the right location, and not necessarily associated with ENSO, can significantly influence USWC conditions and enhance their predictability.

## Introduction

**Figure 1:**
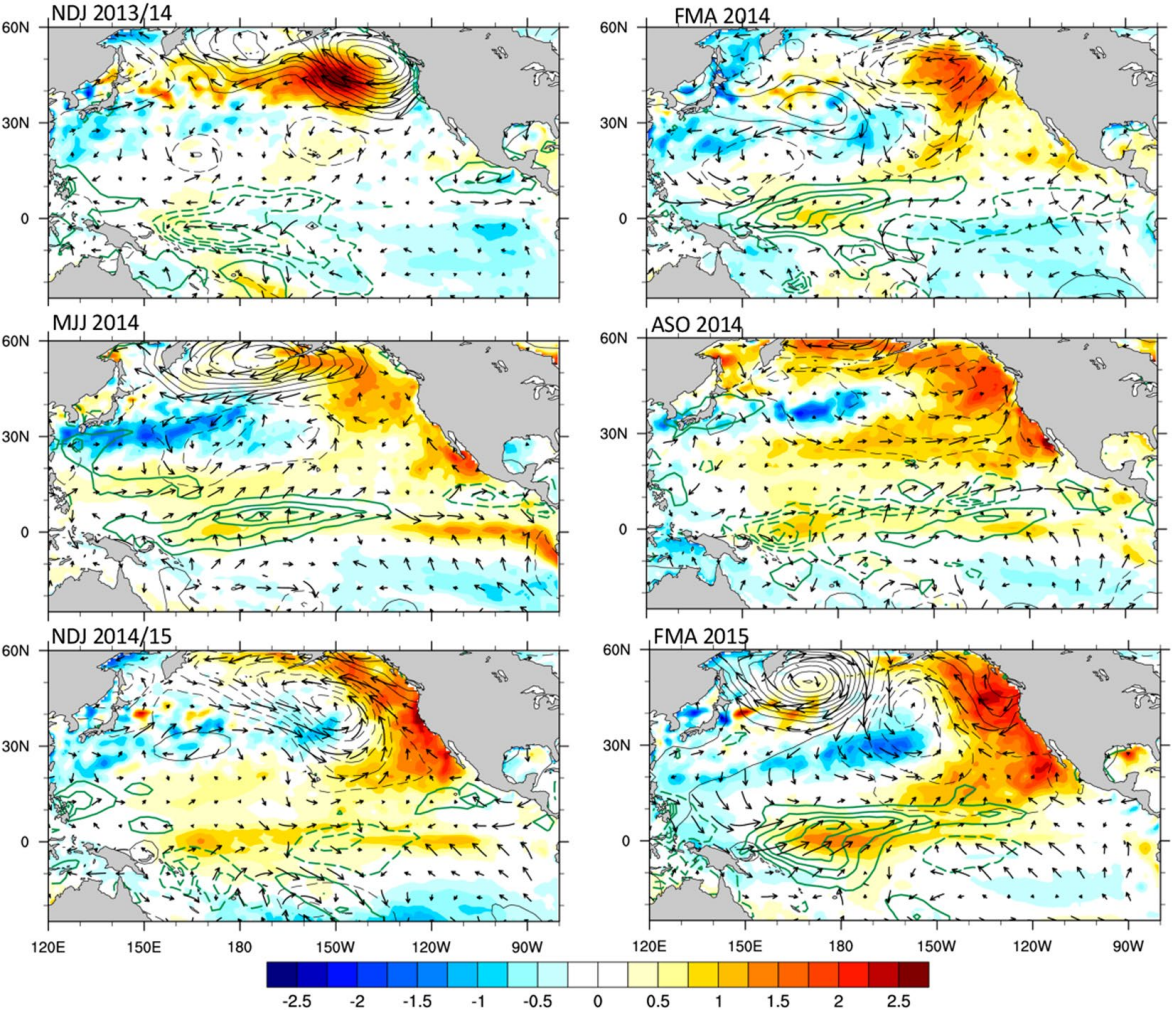
Northeast Pacific “marine heat wave”. Evolution of the SST (shading), SLP (black contours, negative values dashed), precipitation (green contours, negative values dashed) and surface winds (arrows) anomalies, from NDJ 2013/14 to FMA 2015. Contour intervals for SLP and precipitation are 0.8 mbar, and 0.8 mm/day, respectively. SST units are °C.

During the winter of 2014/15, the US west coast (USWC) experienced record high temperatures extending from Baja California to the Gulf of Alaska. This unprecedented warming of as high as 3 °C in some locations^1^, had profound impacts on the California Current System (CCS) and Gulf of Alaska marine ecosystems across multiple trophic levels, whose extent and societal consequences are still being investigated. The warming along the USWC was preceded during the winter of 2013/14 by another unprecedented offshore warming in the Gulf of Alaska, which was termed “the Blob” by Bond *et al*.^2^ because of its intensity and spatial pattern. The sustained warming in the northeast Pacific due to the combination of the Blob in 2013/14 and the USWC warming the following year, as illustrated in Fig. 1, provides a compelling example of extreme marine conditions that have become known as “marine heat waves”^3^. Global warming can apparently increase the likelihood of such extremes^4^. However, natural climate conditions could also have contributed to the sequence of events that occurred over the 2013–15 period. Identifying such conditions is a major goal of this paper.

It is well known that interannual sea surface temperature (SST) variations in the tropical Pacific associated with the warm (El Niño) and cold (La Niña) phases of the El Niño Southern Oscillation (ENSO) exert a strong influence on the USWC SSTs through both oceanic and atmospheric pathways^5^. During El Niño events, downwelling oceanic equatorial Kelvin waves propagate eastward to the eastern ocean boundary, and then continue poleward along the west coasts of both Americas as coastally-trapped Kelvin waves, deepening the thermocline, reducing upwelling and creating warmer than average coastal conditions. Opposite-signed oceanic effects occur during La Niña events. ENSO events also influence remote regions through atmospheric pathways, specifically through SST-forced atmospheric waves. In particular, ENSO affects the position and strength of the Aleutian Low (AL)^6^, which becomes stronger and is displaced eastward during El Niño events. The stronger AL is associated with an atmospheric cyclonic circulation which weakens the equatorward along-shore winds, causing reduced upwelling and warmer conditions along the coast. However, only weak warming occurred in the equatorial Pacific during 2014 (Fig. 1), as the anticipated strong 2014/15 El Niño failed to materialize, causing doubt on the role of the tropical Pacific and ENSO in that year’s USWC warming.

**Figure 2:**
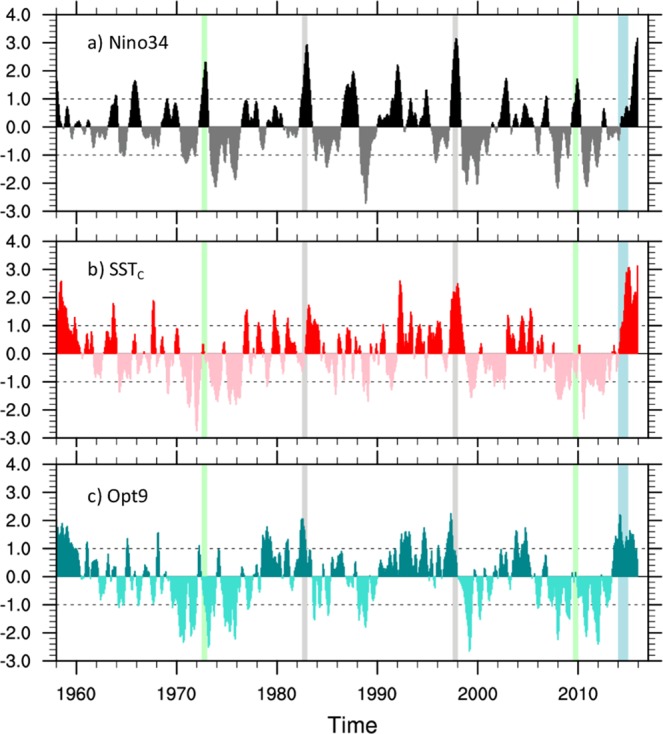
Comparison of relevant time series. (**a**) Time series of the Niño3.4 index (average SST anomaly in the region 5°S–5°N, 150°W–90°W) from January 1958 to December 2015. (**b**) SST anomaly index along the USWC (SST_C_) computed as the SST anomaly averaged in the strip 30°N to 55°N and the coast to approximately 300 km offshore. (**c**) Index describing the evolution of the sensitivity pattern in Fig. 3a. This sensitivity pattern is obtained using equation 1 with a lead time τ = 9 months. The associated index (Opt9) is calculated by projecting SST and SSH anomaly fields onto the sensitivity pattern. Thus, the values of Opt9 at each time correspond to the uncentered pattern correlation of the observed SST/SSH fields with the sensitivity pattern. Gray vertical bars identify two El Niño events (1982/83 and 1997/98) that were associated with large values of SST_C_, light green bars indicate two El Niño events (1972/73 and 2009/10) that corresponded to very small values of SST_C_, and the light blue bar highlights the 2014 case, where strong warming along the USWC was associated with small values of the Niño-3.4 index.

The relationship between ENSO and USWC conditions is illustrated by the relatively good correspondence between the Nino3.4 index (average SST anomalies in the area 5°S–5°N, 120°–170°W, Fig. 2a), a commonly used metric of ENSO, and SST anomalies along the USWC (average SST in the strip 30°N to 55°N and the coast to ~300 km offshore, Fig. 2b). The correlation coefficient between the two time series is 0.52 when the Nino3.4 index leads the USWC index by ~2 months. Visual inspection of the time series shows that the strong El Niño events of 1982/83 and 1997/98 were associated with large SST anomalies along the USWC persisting a few months after the peak of each event (light gray shading), supporting the idea that intense warming in the Niño3.4 area can produce a large response along the USWC. However, other El Niño events (indicated by the light green shading), including the relative strong El Niño of 1972/73, only produced a very small USWC warming. Conversely, the very weak tropical warming during the winter of 2014/15 was associated with a major marine heat wave along the USWC that year (light blue shading).

In this study, we would like to first address the fundamental question of why some El Niño events have a very small impact on the USWC. Is that due to their spatial pattern, intensity, time evolution, or all of these factors? A second question is why some weak El Niño events, like the one in 2014, can have a large impact. To address both questions, we need to clarify how the tropical Pacific can optimally influence the USWC, and consider the possibility that the optimal tropical SST anomaly pattern for forcing large USWC anomalies may not coincide with a canonical El Niño pattern. For example, Barsugli & Sardeshmukh^7^ determined the tropical SST pattern that is most effective in forcing the Pacific North American (PNA) mode of variability. This “sensitivity pattern” was determined through ensembles of atmospheric general circulation model simulations forced by small patches of SST anomalies prescribed at regularly spaced locations over the tropical Indo-Pacific domain. The results showed that the area that is most effective in forcing the PNA is the central-western Pacific, not the eastern tropical Pacific where the SST anomalies associated with ENSO events typically have the largest magnitudes. Thus, only ENSO events with a large projection on the sensitivity pattern found by Barsugli and Sardeshmukh^7^ can be expected to have a large influence on the PNA. Here we are interested in a similar question as in Barsugli & Sardeshmukh^7^, but our target quantity is the SST anomaly along the USWC instead of the PNA mode. Additionally, unlike Barsugli & Sardeshmukh^7^, we use a statistical approach to determine the sensitivity patterns, as described in the Methods section. After identifying the sensitivity patterns in the tropical Pacific, we examine the large-scale conditions associated with those patterns and discuss the reasons why the influence of El Niño on the USWC is event-dependent. We also discuss how the distinctive evolution of the North Pacific atmospheric and oceanic fields preceding the mature El Niño phase may play a key role in the El Niño impact on USWC marine conditions.

## Tropical Sensitivity Patterns

**Figure 3:**
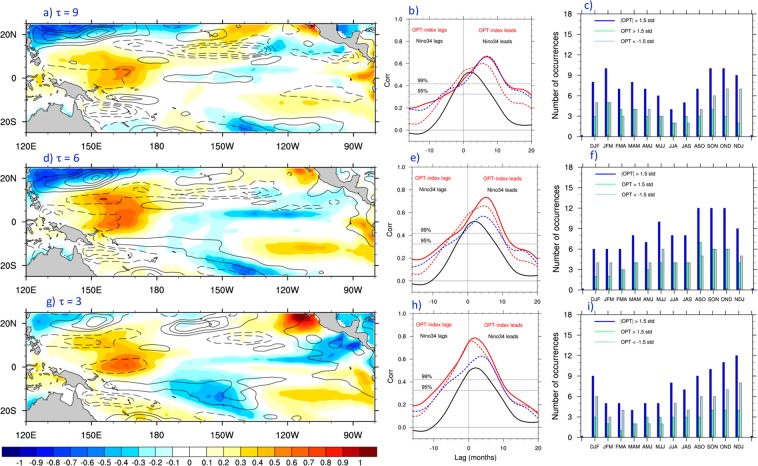
Sensitivity patterns. The left panels show the SST/SSH sensitivity patterns obtained for τ = 9 (**a**), 6 (**d**) and 3 (**g**) months using equation (1). SST anomalies are shaded, while SSH anomalies are contoured. Units are arbitrary. SSH contour interval is 0.2. Middle panels show the lag-correlation of the indices (optimal indices) associated with the sensitivity patterns to the left with the SST_C_ index (red lines) for τ = 9 (**b**), 6 (**e**) and 3 (**h**) months. The lag correlation obtained with the three indices is compared with the lag-correlation between the Niño3.4 index and SST_C_ (black line), as well as with the lag-correlations obtained using only SST in the tropical state vector (red dashed line), and using a meridionally narrower (15°S–15°N) tropical domain (blue dashed line). The optimal indices and the Niño3.4 index lead SST_C_ for positive lags and lag it for negative lags. The values of the correlations corresponding to the 95% and 99% statistical significance levels are indicated by the dotted lines. The right panels provide a measure of the seasonality of the sensitivity patterns on the left. Green bars indicate the number of times the optimal index exceeds 1.5 standard deviations (indicating a strong projection of the SST/SSH anomaly fields at that time on the corresponding sensitivity pattern) in each season, light blue bars indicate the number of times the index is less than −1.5 its standard deviation in each season, and the blue bars are the sum of the previous two cases (|OPT| > 1.5 std).

For our purposes here, we characterized tropical oceanic conditions in terms of SST and sea surface height (SSH). The latter quantity provides information about ocean memory and allows the determination of tropical conditions that are better precursors of SST anomalies along the USWC. We used the SST and SSH fields from the European Centre for Medium-Range Weather Forecasting (ECMWF) Ocean Reanalysis System 4 (ORAS4)^8^ during the period January 1958 to December 2015, as described in the Methods section. The sensitivity patterns were determined using the approach of Capotondi & Sardeshmukh^9^ (see Methods) for lags τ = 9, 6, and 3 months (SP9, SP6, and SP3), which are shown in Fig. 3. For the lead time of 9 months (Fig. 3a), the sensitivity pattern reveals a large positive SST “center of action” in the western equatorial Pacific between 150°E and 180°E. Positive SST anomalies that extend southwestward from the northern boundary of the domain toward the equator are also seen between 130° and 160°W. These anomalies are similar in structure to the North Pacific Meridional Mode (NPMM), an SST anomaly pattern that can promote the development of El Niño events^10^. The positive anomalies in the southeast Pacific, on the other hand, are reminiscent of the South Pacific Meridional Mode (SPMM), another precursor proposed by Zhang *et al*.^11^ for the development of El Niño events. And finally, the large negative anomalies in the northwestern corner of the domain capture some aspects of the Northwest Pacific ENSO precursor^12^. The SSH anomalies display the largest loadings off the equator and are likely associated with westward propagating off-equatorial oceanic Rossby waves, whose excitation and westward propagation are part of the tropical Pacific adjustment process at interannual and decadal timescales^13–16^. Indeed, while the SST anomalies associated with the NPMM can propagate southwestward all the way to the equator through the wind-evaporation-sea surface temperature (WES) feedback^17^, and influence the equatorial winds, the subtropical wind stress anomalies associated with the NPMM can also influence the equatorial thermocline through off-equatorial Rossby wave excitation^13,14^, as well as meridional equatorward oceanic transport^18^. In particular, Rossby waves in the 10°–15°N latitude band have been shown to reach the western ocean boundary, continue along the boundary to the equator as coastal Kelvin waves, and then propagate eastward along the equator as equatorial Kelvin waves, altering the depth of the thermocline, and possibly inducing equatorial SST anomalies several months after the wave excitation in the northern tropics^14^.

An amplitude index of the sensitivity pattern in Fig. 3a was computed by projecting the ORAS4 SST and SSH fields at each time step on the SP9 SST/SSH patterns. Thus, the value of the index at time t is the uncentered pattern correlation of the SST/SSH fields at that time and the sensitivity pattern. The resulting index, which we term “Opt9”, is shown in Fig. 2c. Comparison with the Niño3.4 index shows that Opt9 attains large values during several El Niño events, including the 1982/83 and 1997/98 events, indicating that those events were characterized by a large projection on the SP9 pattern. On the other hand, the El Niño events associated with little USWC warming have relatively small projections on the sensitivity pattern, as indicated by the low values of the index during those years. The index also shows large values during 2014, indicating that in spite of only a weak El Niño warming, the tropical Pacific conditions during 2014 were conducive to a large USWC warming signal. Opt9 has larger correlations with SST_C_ than the Niño3.4 index at almost all lags (Fig. 3b). The maximum correlation of 0.66 is achieved when Opt9 leads SST_C_ by ~7 months. Thus, Opt9 is a better predictor of USWC warming than canonical El Niño indices at a longer lead time.

To examine the seasonality of the sensitivity pattern in Fig. 3a we consider the frequency of occurrence of Opt9 values above (below) a certain threshold, chosen to be 1.5 (−1.5) standard deviations. The results are shown in Fig. 3c. For positive values (bright green bar), the largest number of occurrences is in JFM, while the negative pattern tends to occur more frequently during late Fall/early Winter (October to January, light blue bar). As a result, the number of occurrences of either the positive or negative phase of the sensitivity pattern maximizes during fall to late winter (dark blue bar in Fig. 3c).

Since the sensitivity pattern for τ = 9 months includes some features of extra-tropical ENSO precursors, one may wonder if the sensitivity patterns for shorter lead times are more similar to a fully developed ENSO pattern. However, the sensitivity patterns for τ = 6 and 3 months (Fig. 3d,g) also do not look like the canonical ENSO pattern. Instead, features already present in the sensitivity pattern for τ = 9 months, including the positive anomalies in the western Pacific, in the southeast Pacific, and near Baja California, are also present at shorter lead times. The lag correlations of the associated indices (Opt6 and Opt3) with the SST_C_ index increase with decreasing lead time, achieving maximum values of 0.74 and 0.79 when Opt6 and Opt3 lead SST_C_ by 5, and 2 months, respectively (Fig. 3e,h). These sensitivity patterns occur more frequently toward the end of the calendar year (Fig. 3f,i), with the maximum number of occurrences during September to December for the 6-month pattern and during NDJ for the 3-month pattern.

The importance of including SSH in the tropical state vector is highlighted by the comparison with lag-correlations obtained when only SST information is used (Fig. 3b,e–h, red dashed lines). Maximum correlations are lower than those obtained with the inclusion of SSH, and tend to be achieved at slightly shorter lead times, consistent with the findings of Newman *et al*.^19^. Given the presence of features associated with extra-tropical ENSO precursors in the sensitivity patterns near the northern boundary of the domain, the role of these components relative to the equatorial anomalies may be questioned. Calculations repeated over a meridionally narrower domain (15°S–15°N) yield almost unchanged lag-correlations for τ = 9 months, but lower correlations at τ = 6 and 3 months (Fig. 3b,e, and h, blue dashed lines), suggesting that at shorter lead times the presence of the extra-tropical precursors, and their association with specific North Pacific atmospheric fields, as discussed in section 3, may play a relatively more important role in the establishment of the coastal SST anomalies.

## Large-scale Conditions Associated with the Sensitivity Patterns

**Figure 4:**
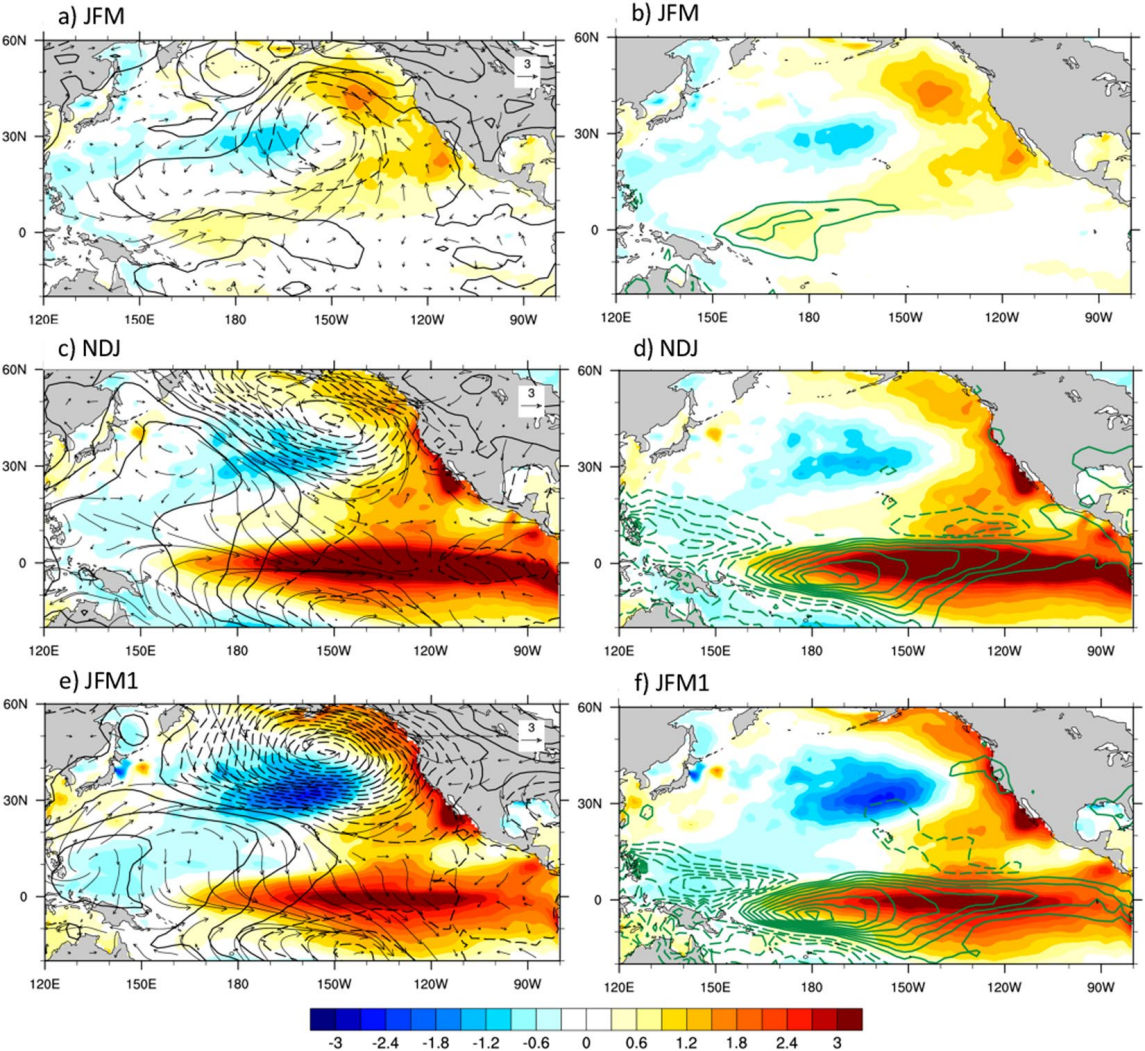
Composite evolution of optimal oceanic and atmospheric conditions. Composites (positive minus negative occurrences) of SST (shading), SLP (contours) and surface wind (arrows) anomalies on the left, and SST (shading) and precipitation (green contours) anomalies on the right, based on the sensitivity pattern obtained for τ = 3 months (Fig. 3g). The largest number of occurrences of this pattern is in NDJ. Composites for this season are shown in (**c**) and (**d**), while (**a**) and (**b**) show the composites for the preceding JFM, and (**e**) and (**f**) the composites for the following JFM seasons. Contour intervals are 0.3 °C for SST, 1 mbar for SLP, and 1 mm/day for precipitation.

What is the physical meaning of the sensitivity patterns? And what are the large-scale climate conditions associated with them? To address these questions, we constructed composites of various climate variables during times of large sensitivity index values. Focusing on SP3, we computed composites of SST, SLP, surface winds and precipitation for the cases in which Opt3 was larger than 1.5 standard deviations or smaller than −1.5 standard deviations during NDJ, and considered the positive minus negative cases. Results are shown in Fig. 4c,d. The composite SST anomaly pattern resembles a mature El Niño event. Indeed, as noted before, many El Niño events have a large projection on the sensitivity pattern. The SLP pattern (Fig. 4c) shows a deepened and eastward displaced Aleutian Low, with associated counterclockwise wind anomalies, which weaken the winds that favor coastal upwelling. Reduction of upwelling and turbulent heat fluxes is associated with warm conditions along the USWC. Precipitation anomalies (Fig. 4d) show large positive values centered around the dateline, with positive anomalies extending toward the eastern side of the basin. Negative anomalies are seen in the far western Pacific and are associated with the eastward displacement of convection along the equator.

In the following JFM season, El Niño conditions start to decay in the tropical Pacific, but the SLP, surface wind and precipitation anomalies intensify further, resulting in enhanced warming along the USWC (Fig. 4e,f). It is interesting to examine the basin-wide conditions prior to the development of SP3. Figure 4a,b show the composite SST, SLP, surface winds and precipitation during the JFM season preceding the development of the tropical sensitivity pattern. SLP anomalies over the North Pacific exhibit a negative anomaly centered at 30°N, 150°W, which is associated with a cyclonic circulation that opposes the climatological trade winds in the subtropics. This reduction of the trade wind strength results in decreased heat loss by the ocean through turbulent heat fluxes and the development of the positive subtropical SST anomalies associated with the NPMM^10^. The low SLP center is indeed similar to the southern lobe of the North Pacific Oscillation (NPO)^20,21^ in its negative phase. The NPO, the second leading mode of North Pacific SLP anomalies, has been associated with the atmospheric forcing of the North Pacific Gyre Oscillation (NPGO)^22^, and its southern lobe is considered as the atmospheric forcing of the NPMM. The anomalous winds in Fig. 4a are also conducive to the development of positive SST anomalies in the Gulf of Alaska and off Baja California. The anomalous atmospheric circulation pattern also exhibits westerly wind anomalies in the western equatorial Pacific, responsible for the development of a positive SST anomaly in that area. Notice that this SST anomaly is associated with a co-located precipitation anomaly (Fig. 4b), an indication of atmospheric diabatic heating which may be a driver of atmospheric teleconnections. Figure 4a,b depict a distinctive evolution of the atmospheric and oceanic fields with both extra-tropical and equatorial signatures. While the extra-tropical component may precondition the USWC region by creating warm conditions and a deeper thermocline, the development of tropical SST anomalies may establish the atmospheric teleconnections that sustain the extra-tropical atmospheric circulation and support the enhancement of the USWC warming.

## Why Do Some El Niño Events not Have any USWC Warming?

**Figure 5:**
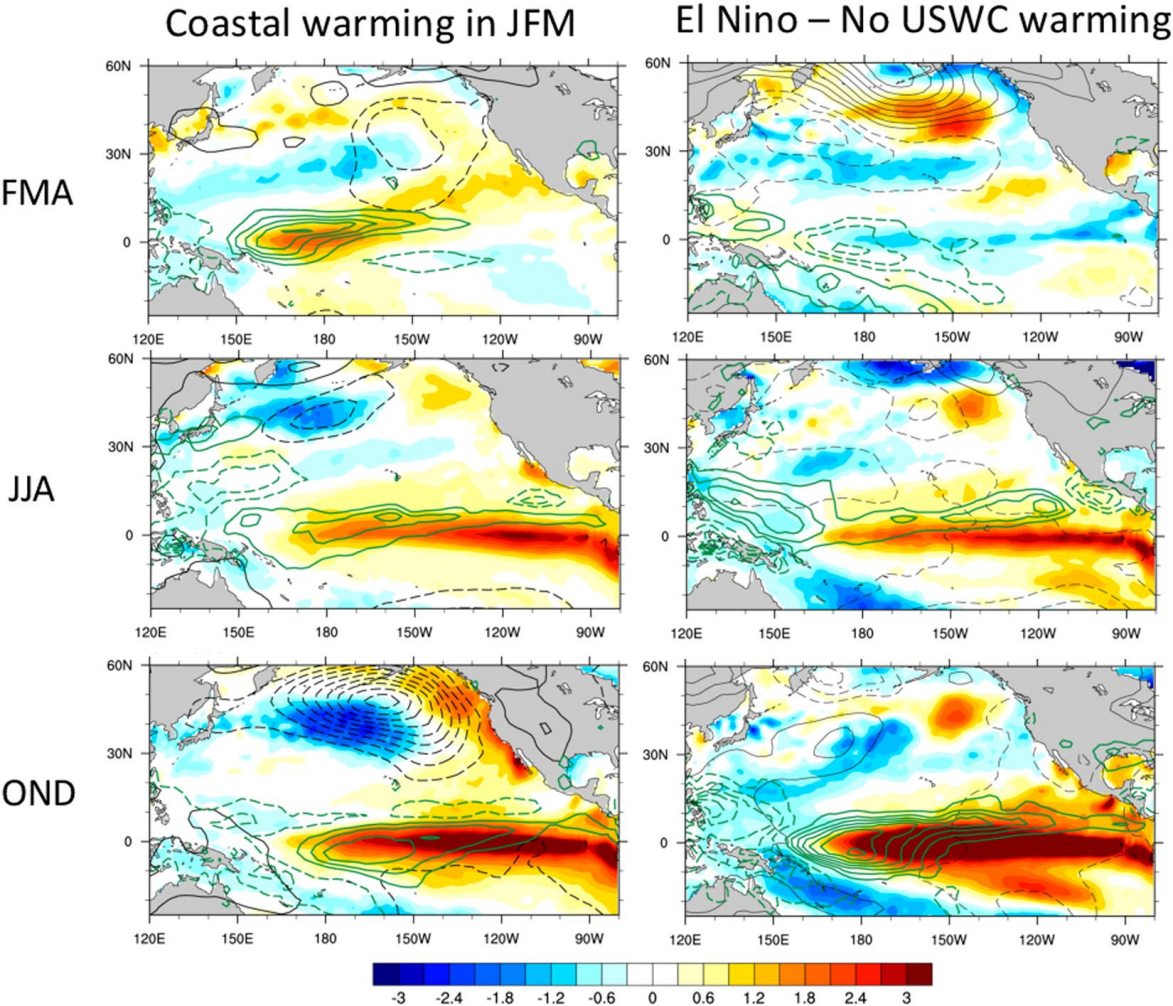
USWC warming vs. no-USWC warming. Comparison of the composite evolution of SST (shading), SLP (black contours, negative values dashed) and precipitation (green contours, negative values dashed) anomalies during El Niño events with no USWC warming (right panels) and for the cases of USWC warming during JFM, a season characterized by the largest USWC SST variance (left panels). El Niño events with no USWC warming were identified by requiring that the Niño3.4 index exceeds 1 standard deviation during DJF, but the SST_C_ index is below 1 standard deviation during JFM of the same winter. The fields are displayed for the FMA, JJA and OND seasons prior to the Niño3.4 and SST_C_ maxima. Contour intervals are 0.1 °C for SST, 0.5 mbar for SLP, and 0.5 mm/day for precipitation.

The sensitivity index has helped us identify key elements of the evolution of the climate system in the tropical and North Pacific that are conducive to USWC warming. We now complement that analysis with the direct examination of the composite evolution of SST, SLP, and precipitation fields during cases of USWC warming, and contrast that evolution with that obtained during the El Niño events that do not produce warming along the USWC. The SST along the USWC achieves its largest standard deviation in late Winter (JFM, not shown). For the cases when our SST_C_ index is larger than one standard deviation during JFM, we composite the conditions during FMA, JJA, and OND of the previous year (Fig. 5, left panels). The results show an evolution very similar to that found using the sensitivity index. During FMA of the previous year, there is a weak anomalous SLP low centered around 30°N, subtropical SST anomalies resembling the NPMM and anomalous SSTs in the western equatorial Pacific which are co-located with precipitation anomalies. This pattern also bears some similarities with the El Niño Modoki pattern described by Ashok *et al*.^23^, although the latter tends to be more prominent during boreal Summer. An El Niño event develops in the equatorial Pacific in the subsequent seasons. During OND, the equatorial warming is associated with a more intense and eastward displaced Aleutian Low and enhanced positive SST anomalies along the USWC.

We next consider the SST, SLP, and precipitation evolution during El Niño events (identified using the criterion that the Niño3.4 index in DJF is larger than one standard deviation) that are not accompanied by USWC warming (SST_C_ < 1 standard deviation in the following JFM, Fig. 5, right panels). These events include the 1965/66, 1972/73, 1986/87, 1994/95, and 2009/10 El Niños. It is interesting that some of these events, especially the 1972/73 case, were strong events, but their amplitude did not translate into a large impact on the USWC. In contrast to the case of USWC warming, these events exhibited cold conditions along the equator during the preceding FMA season, likely associated with a lingering La Niña from the previous year, and approximately co-located with negative precipitation anomalies. Negative SST anomalies were present along the USWC during FMA, and although the tropical Pacific conditions evolved to a mature El Niño state by OND, no typical Aleutian Low response to El Niño developed in the North Pacific, and no warming along the USWC occurred.

This analysis not only confirms the results based on the sensitivity indices, but also highlights the importance of the diversity in the details of El Niño development, both in the extra-tropics and along the equator, for USWC impacts. The North Pacific SLP and wind anomalies associated with the sensitivity indices are themselves conducive to warming along the USWC, thus preconditioning that area through local dynamical and thermodynamical processes. However, the North Pacific conditions preceding USWC warming are also associated with warm anomalies in the central/western Pacific, as shown by the 9-month sensitivity pattern (Fig. 3a), and by the composite fields in Fig. 4a,b. These SST anomalies occur concurrently with the development and evolution of the NPMM, as noted in previous studies^24^, and are located in a very sensitive area of the equatorial Pacific. Indeed, SST anomalies in the central/western Pacific have been shown to be especially effective in forcing the PNA^7^ and in producing a stronger Aleutian Low and USWC response, relative to anomalies in the eastern equatorial Pacific^25^. They can themselves force SST anomalies like those associated with the NPMM^24^, and likely sustain North Pacific anomalous atmospheric conditions like those that have occurred during 2013–2015^26^. These results highlight a strong interplay between North Pacific and tropical Pacific conditions whose exact dynamics deserve further explorations in future studies.

## Summary and Discussion

Our study has identified SST and SSH conditions in the tropical Pacific that are most conducive to large SST anomalies along the US West Coast. These optimal precursors or “sensitivity patterns” do not exactly coincide with canonical ENSO patterns - although mature ENSO events may have a large projection on them – and instead highlight the important role of some precursors of ENSO events, in particular, the North Pacific Meridional Mode (NPMM). The NPMM consists of SST anomalies extending southwestward from Baja California toward the equator, which result from the weakening of the trade winds associated with the North Pacific Oscillation (NPO). The latter consists of a north-south sea level pressure (SLP) dipole in the North Pacific, with the northern pole centered approximately over Alaska and the southern pole reaching all the way to Hawaii. The SLP anomalies of the southern lobe of the NPO modulate the trade winds creating subtropical wind anomalies which can influence the tropical Pacific in several ways. They produce SST anomalies which can feedback to the winds, resulting in a southwestward propagation of the anomalies^10,27^. These wind anomalies can also force oceanic Rossby waves which propagate to the western ocean boundary and continue along the boundary to the equator as coastal Kelvin waves^13,14^; and finally, the subtropical wind stress curl anomalies associated with the southern lobe of the NPO can alter the interior equatorward ocean transport, producing a “Tropical Wind Charging” (TWC) of the equatorial thermocline^18^. The phase of the NPO with low SLP anomalies in its southern lobe tends to be accompanied by warm anomalies in the Gulf of Alaska, an expression of the North Pacific Gyre Oscillation^22^, and favors warming along the coast, thus preconditioning that area towards positive SST anomalies.

These features of the North Pacific atmospheric circulation and of its oceanic expression emerge from our composite analysis of the basin-scale climate conditions associated with the sensitivity pattern for three months lead time. In addition, our analysis shows that besides the subtropical surface wind anomalies associated with the development of the NPMM, westerly wind anomalies were also present along the equator several months prior to the development of large SST anomalies along the USWC. These wind anomalies resulted in western equatorial warming and co-located precipitation anomalies which are likely triggers of teleconnections to the North Pacific. At shorter lead times, the signature of the NPMM is less evident, but the western tropical Pacific SST anomalies remain the dominant component of the sensitivity patterns. The presence of these anomalies in the western Pacific may be responsible for the deepening and southeastward displacement of the Aleutian Low, which would directly affect the USWC through reduced upwelling and reduced heat loss via turbulent heat fluxes^28^.

This sequence of events is consistent with the findings of Di Lorenzo & Mantua^29^, who explained the evolution and persistence of the 2013–2015 marine heat wave in the northeast Pacific as the result of a shift from NPO forcing and Gulf of Alaska warming during the Winter of 2013/14, to the development of a stronger and southeastward displaced Aleutian Low in response to the warm equatorial Pacific conditions. Although these warm conditions could barely qualify as an El Niño event, their location in the central/western equatorial Pacific made them particularly effective for the excitation of atmospheric teleconnection affecting the PNA^7,25^. Although ENSO events exhibit a variety of spatial patterns^30^, with central Pacific events being generally weaker than eastern Pacific events, even small SST anomalies in the central/western Pacific, where they are superimposed to the “Warm Pool” background conditions may be as or more effective than larger anomalies in the eastern Pacific “Cold Tongue” region.

The northeast Pacific marine heat wave is one aspect of a broader northern Hemisphere climate state during 2013/15 that also included persistent dry conditions in California, as well as intense cooling over the northeastern US. A persistent northeast Pacific SLP ridge has been related to the lack of precipitation over California as well as to the cold conditions further east. Some studies have attributed the persistence of that ridge to the influence of the NPO during 2013^31^, and to the presence of the Northwest Pacific ENSO precursor^32^. The tropical Pacific influence has also been considered crucial for the establishment and persistence of these SST and atmospheric circulation patterns^26^. The interplay between the North Pacific atmospheric circulation and tropical Pacific highlighted in these previous studies is consistent with our findings, obtained through a very different approach. These tropical extra-tropical interactions have also been invoked as a primary mechanism for energizing Pacific decadal variability^33^. In addition, the NPO/ENSO teleconnection is projected to increase under global warming, and result in an intensification of the Pacific climate variance^34^.

Although ENSO precursors appear to play an important role in the development of USWC warming, not all El Niño events display the same evolution. In particular, events with late onset and/or with SST anomalies originating in the eastern Pacific and propagating westward do not produce warm conditions along the USWC. These events are not associated with the same North Pacific preconditioning and have a small projection on our sensitivity patterns. Thus, our analysis allows us to distinguish El Niño events that produce USWC warming from the events that do not. The relative importance of the local preconditioning of the USWC by the North Pacific atmospheric circulation vs. the equatorial Pacific influence is an aspect of our study that needs to be further elucidated. In other words, are the SST anomalies in the western equatorial Pacific actively driving the North Pacific air-sea interactions responsible for the coastal marine warming, or are they only passively concurrent with those interactions? While previous studies^7,24^ have shown an active role for SST anomalies in the central/western equatorial Pacific in forcing the PNA and the NPMM, additional sensitivity experiments in coupled model contexts would be very useful to further elucidate the role of the tropical Pacific in the development and evolution of the northeast Pacific marine heat wave during 2013–2016. Finally, having identified the atmospheric and oceanic conditions conducive to USWC warming, future work is also required to examine whether these conditions may become more likely to occur in a warming climate.

## Methods

The SST/SSH fields were obtained from the European Centre for Medium-Range Weather Forecasting (ECMWF) Ocean Reanalysis System 4 (ORAS4)^8^ for the period January 1958 to December 2015. ORAS4 has a horizontal resolution of 1° in the extra-tropics, with higher meridional resolution in the tropics up to a value of 0.3° at the equator. It has 42 vertical levels, with 18 levels in the upper 200 m, and it is forced at the surface by heat, freshwater, and momentum fluxes from a suite of ECMWF atmospheric reanalysis products. The ORAS4 output was obtained from http://icdc.cen.uni-hamburg.de/projekte/easy-init/easy-init-ocean.html.

To examine the large-scale fields associated with the development and evolution of the tropical sensitivity patterns we used SLP and surface winds from the National Center for Environmental Prediction (NCEP)/National Center for Atmospheric Research (NCAR) reanalysis^35^, and NOAA precipitation reconstruction^36^ over the same period (1958–2015). Both data set are available from https://www.esrl.noaa.gov/psd/data/gridded.

To determine the tropical ocean conditions that optimally force SST anomalies along the USWC at different lead times we adopted the statistical approach used by Capotondi & Sardeshmukh^9^ to determine the optimal precursors of different types of ENSO events. In this view, the time series of SST along the USWC (Fig. 1b) at lag τ, *y* (τ), is related to the tropical Pacific (25°S–25°N) state vector ***x*** (0) at the initial time *t* = 0 through an operator ***H*** (τ) according to:

1 $$y(\tau )={\boldsymbol{H}}(\tau ){\boldsymbol{x}}(0)+{\boldsymbol{\varepsilon }},$$

where **ε** represents the unpredictable noise residual. The tropical Pacific conditions, as encapsulated in the anomaly state vector ***x***, are described in terms of SST and SSH. Both fields are available in ORAS4, ensuring dynamical consistency. The inclusion of tropical SSH information helps identify sensitivity patterns that are better predictors of SST_C_ than those obtained using only tropical SSTs. Since SSH is dynamically linked to thermocline depth, the SSH fields contain information about subsurface ocean processes responsible for the ocean adjustment and memory^19^. When only SST is used in the state vector the resulting SST sensitivity patterns are very similar to those obtained with both SST and SSH, and correlations of the associated indices with SST_C_ are still higher than those obtained using the Niño3.4 index (Fig. 3, middle panels). However, they are not as high as those achieved with the inclusion of SSH in the state vector. The lead times of the largest correlations are also slightly shorter when only SST is considered.

The SST and SSH fields were smoothed in time with a 3-month running mean and linearly detrended prior to projecting them onto their leading empirical orthogonal functions (EOFs), a step used to reduce the number of degrees of freedom. We have retained 20 SST and 20 SSH EOFs, which explain 91% and 87% of the variance of the corresponding fields. ***H*** (τ) is computed through multiple linear regressions, and the optimal initial state ***x*** (0) is determined as the leading right singular vector of ***H***. Unlike the lag-correlations of *y* with SST (or SSH) in the tropical Pacific at each grid point, which would produce patterns reflecting the inter-dependence of the tropical SST (SSH) evolution due to ENSO, the approach encapsulated by (1) removes that inter-dependence and yields the patterns that are only related to *y* at the given lag. The methodology has been cross-validated by withholding independent 10-yr segments from the 58-yr data record and repeating the calculation on the remaining 48 years. The resulting sensitivity patterns remain very similar to those obtained with the full record. The lag-correlations of the corresponding optimal indices with the SST_C_ index are slightly lower, especially for the 9-month lead time (they are almost unchanged for τ = 3 months), but the maximum correlations remain larger than those obtained with the Niño-3.4 index, and are still achieved at longer lead times.
